# The association between albumin and mortality in patients with acute kidney injury: a retrospective observational study

**DOI:** 10.1186/s12882-023-03323-x

**Published:** 2023-11-09

**Authors:** Kaibi Yang, Nan Yang, Wenbo Sun, Limiao Dai, Juan Jin, Juan Wu, Qiang He

**Affiliations:** 1 Jinzhou Medical University, Jinzhou, Liaoning, 121001 China; 2 Urology & Nephrology Center, Department of Nephrology, Zhejiang Provincial People’s Hospital (Affiliated People’s Hospital), Hangzhou Medical College, Hangzhou, Zhejiang China; 3 Department of Nephrology, the First Affiliated Hospital of Zhejiang Chinese Medical University (Zhejiang Provincial Hospital of Traditional Chinese Medicine), Hangzhou, 310000 Zhejiang China

**Keywords:** Acute kidney injury, Albumin, Prognosis

## Abstract

**Background:**

While the association between decreased serum albumin (ALB) levels and increased risk of acute kidney injury (AKI) is well established, the risk of death among patients with AKI with low serum ALB levels is unclear. We aimed to evaluate the association between serum ALB levels in patients with AKI and mortality, and help guide their clinical management.

**Methods:**

The included patients were those diagnosed with AKI and admitted to Zhejiang Provincial People's Hospital between January 2018 and December 2020. The clinical endpoint was all-cause mortality rate at 90-days and 1-year. Patients were divided into four groups according to the quartiles (Qs) of ALB measurements at admission. Cumulative survival curves were calculated using Kaplan–Meier analysis, and Cox proportional risk models were used to assess the association between serum ALB levels and 90-day and 1-year all-cause mortality.

**Results:**

This study included 740 patients with AKI. Patients with measured ALB values were classified into quartiles: Q1 ≤ 26.0 g/L (*n* = 188); Q2 = 26.1–30.5 g/L (*n* = 186); Q3 = 30.6–34.7 g/L (*n* = 183); Q4 ≥ 34.8 g/L (*n* = 183). Univariate analysis using Cox regression showed that for every 10 g/L increase in ALB, the 90-day and 1-year mortality decreased by 29%. Among the four subgroups, patients with lower ALB levels had a higher risk of death. After adjusting for demographics, comorbid conditions, inflammatory index, and medicine, the lowest ALB quartile (ALB < 26 g/L) was associated with increased risk of 90-day mortality (hazard ratio [HR], 1.76; 95% confidence interval [CI], 1.30 to 2.38, *P* < 0.001) and 1-year all-cause mortality (HR, 1.79; 95% CI, 1.33 to 2.41, *P* < 0.001).

**Conclusions:**

ALB levels in patients with AKI were significantly correlated with prognosis, and the higher the level, the better the prognosis. Compared to patients with ALB ≥ 34.8 g/L, patients with 26.1 g/L < ALB ≤ 30.5 g/L had an increased risk of 90-day and 1-year all-cause mortality of approximately 40%, and patients with ALB ≤ 26.0 g/L had an increased risk of 90-day and 1-year all-cause mortality of approximately 76% and 79%, respectively.

## Introduction

Acute kidney injury (AKI) is a major complication in hospitalized patients and is closely related to the short- and long-term incidence rates and mortality of severe patients [1]. AKI is an independent risk factor for progression to chronic kidney disease (CKD) [2, 3], and it has a high incidence rate, mortality, and treatment costs, which imposes a huge economic burden on families and society [4]. Ensuring adequate hydration and volume status is essential for preventing and treating AKI [5], and the 2012 Kidney Disease: Improving Global Outcomes (KDIGO) AKI guidelines suggest initiating Renal Replacement Therapy (RRT) in the presence of life-threatening changes in fluid, electrolyte, and acid–base balance. At present, the treatment of AKI remains challenging, and no specific drugs are available [6].

Albumin (ALB) is an important indicator of human nutritional status and is one of the most critical proteins in human plasma because it maintains plasma colloid osmotic pressure, engages in material transport in blood circulation, and facilitates communication among the intracellular, extracellular, and tissue fluids [7]. Recent studies have shown that low ALB levels are an independent risk factor for AKI [8]. It is also a risk factor for death in hospitalized patients [9, 10]. Our aim was to investigate whether ALB levels have an impact on the prognosis of patients with AKI.

## Materials and methods

### Study design and setting

This retrospective study included all patients admitted with AKI to Zhejiang Province People’s Hospital between 2018 and 2020. Patients were initially selected from the hospital electronic records using the International Classification of Diseases, Tenth Revision codes (N17) but were only included after confirming that they met the KDIGO criteria for AKI [11]. Patients with the following conditions were excluded from this study: (1) age < 18 years, (2) length of hospitalization < 48 h, (3) stage 5 CKD or kidney transplantation, and (4) incomplete medical records. For patients with multiple hospitalizations, only the first hospitalization was included in the analysis (as shown in the flowchart). The following variables were included in the current study: (1) baseline demographic data (age and sex) and comorbidities (registered comorbidities included diabetes mellitus, hypertension, CHD (coronary atherosclerotic heart disease), CKD, HF (heart failure), cerebral infarction, cancer, sepsis, and COPD (chronic obstructive pulmonary disease)); (2) laboratory indicators included blood routine, inflammatory index, liver function, kidney function, and electrolytes; and (3) medications used during hospitalization.

Definitions: Hospital-acquired (HA)-AKI and community-acquired (CA)-AKI.

First, AKI was identified based on changes in serum creatinine (Scr) levels during hospitalization. The criteria included the 2012 KDIGO AKI definition: an increase in Scr level by 0.3 mg/dL within 48 h or by 50% within 7 days (not including urine output criteria) [11]. For those who had no repeat Scr assay within 7 days and those recovering from AKI, we expanded the criteria as follows: an increase or decrease of 50% in Scr level during the hospital stay, using the lowest or the highest Scr value during hospitalization as the comparator.

We then identified CA-AKI when patients met any of the following criteria: (1) Scr level was elevated at admission and tended to decline during hospitalization; (2) Scr level was elevated upon admission and continued to increase or remained at a high level during hospitalization, and the Scr value before admission confirmed the presence of AKI; and (3) normal renal function upon admission, an increase in Scr levels, and AKI could then be determined within 24 h of admission [12–14]. For HA-AKI patients who had no reliable Scr record before admission and no evidence of baseline CKD, baseline Scr level was defined as the lowest Scr value that was available within 3 months prior to admission and throughout the hospital stay. For CA-AKI patients who had no reliable Scr record before admission and no evidence of baseline CKD, a back-estimation of the baseline Scr level was performed based on the 4-variable Modification of Diet in Renal Disease (MDRD) study equation with the assumption of an estimated glomerular filtration rate of 75 mL/min/1.73 m^2^, following the recommendations of the 2012 KDIGO AKI clinical practice guidelines [15, 16].

During hospitalization, kidney function and AKI severity were assessed using the KDIGO AKI guidelines [11]. KDIGO classifies severity into three stages based on the increase in creatinine over baseline levels, with stage 1 being the mildest and stage 3 the most severe, and AKI 2 and 3 are collectively referred to as critical AKI. The cohort was followed up for 12 months, and patient survival was tracked through the hospital electronic systems or by telephone.

### Statistics

Patients with measured ALB values were classified into quartiles (Qs): Q1 ≤ 26.0 g/L; Q2 = 26.1–30.5 g/L; Q3 = 30.6–34.7 g/L; Q4 ≥ 34.8 g/L. Participant characteristics were calculated using Qs of ALB. Descriptive analysis was performed to summarize the demographic characteristics and baseline data of the different groups. Mean ± standard deviation (SD) was calculated for continuous variables with normal distribution and median (interquartile distance [IQR]) was calculated for continuous variables with non-normal distribution. Categorical variables are expressed as ratios. Chi-squared, one-way analysis of variance (ANOVA), or Kruskal–Wallis tests were used to test for differences in categorical or continuous factors among the different categories of ALB. Univariate Cox regression was used to evaluate the factors associated with the 90-day and 1-year mortality rates. A multiple multivariate adjustment model was developed to determine the effect of ALB levels on mortality risk. The results are expressed as hazard ratios (HR) and 95% confidence intervals (95% CI). A restricted cubic spline (RCS) regression model was used to further explore the association between ALB levels and outcomes. Kaplan–Meier analysis was used to calculate the cumulative survival curve, and log-rank test was used to evaluate the difference in survival probability between groups. All statistical analyses were performed using SPSS 23.0 (IBM Corp., Armonk, NY, USA), Stata SE16 and R software version 4.3.1. *P*-values < 0.05 were considered statistically significant.

### Outcomes

Patients were followed-up from the time of hospital admission to assess the outcomes. The primary outcome was all-cause mortality at 90-days and 1-year.

**Figure:**
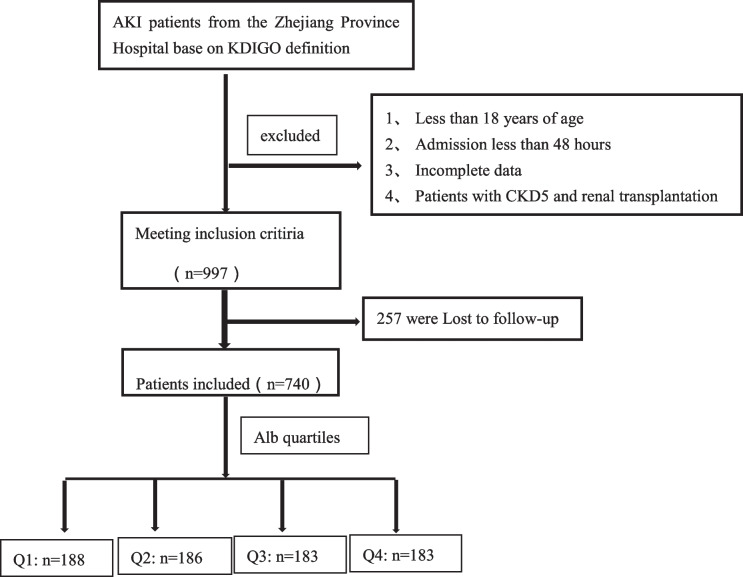
Flow chart

## Results

### Baseline characteristics

Among the 997 patients with AKI during admission, 257 were lost to follow-up or refused follow-up and were excluded; the remaining 740 patients were included (Flow chart). The baseline serum ALB level was 30.4 ± 6.39 g/L. Of the 740 patients, 477 (64.9%) were male, 211 (28.5%) patients had HA-AKI, 60 (8.1%) patients received ALB transfusions before AKI, 83 (11.2%) patients had CKD, 350 (47.3%) patients were treated with continuous renal replacement therapy (CRRT), 431 (58.2%) were treated in the intensive care unit (ICU), and 585 (79.1%) patients had critical AKI.

The baseline characteristics of patients according to the Qs of serum ALB levels are shown in Table 1. Patients with lower ALB levels had a higher proportion of older people. They had higher white blood cells (WBC), red cell distribution width-standard deviation (RDW-SD), C-reactive protein (CRP), and B-type natriuretic peptide (BNP) levels. They also had lower Hb and mean corpuscular hemoglobin concentration (MCHC) levels. Moreover, these patients had a higher proportion of being treated in the ICU and a lower proportion of patients had HA-AKI (*P* < 0.05). There were no significant differences among the groups in Scr (baseline), blood glucose (Glu) level, critical AKI, CKD, and HF patients.

**Table 1:** Baseline characteristics of individuals stratified by quartiles of baseline serum ALB levels

Variable	ALL(*n* = **740**)	≤ 26.0 (*n* = 188)	26.1–30.5 (*n* = 186)	30.6–34.7 (*n* = 183)	≥ 34.8 (*n* = 183)	*P*
Age(years)	71 (57, 82)	73 (62, 83.5)	73 (62.5, 83.5)	75 (61.5, 83.5)	69 (56, 81)	0.006
Sex, Male(%)	477 (64.5)	125 (66.5)	115 (61.8)	119 (65)	118(64.5)	0.81
Comorbidities % (n)						
Diabetes	174 (23.5)	46 (24.5)	39 (21.0)	44 (24.0)	45 (24.6)	0.8
CHD	137 (18.5)	25 (13.3)	30 (16.2)	49 (26.8)	33 (18.0)	0.006
Hypertension	375 (50.7)	77 (41)	94 (50.5)	114 (62.3)	90 (49.2)	0.001
HF	183 (24.7)	43 (22.9)	53 (28.5)	48 (26.2)	39 (21.3)	0.37
CI	131 (17.7)	34 (18.1)	31 (16.7)	31 (16.9)	35 (19.1)	0.92
Cancer	144 (19.5)	37 (19.7)	39 (21)	29 (15.8)	39 (21.3)	0.53
COPD						
Sepsis	283 (38.2)	87 (46.3)	89 (47.8)	52 (28.4)	55 (30.1)	< 0.001
CKD	83 (11.2)	21 (11.2)	19 (10.2)	19 (10.4)	24 (13.1)	0.80
Critical AKI	585 (79.1)	155 (82.4)	145 (78)	139 (76)	146 (79.8)	0.46
Medicine						
PPI	571 (80.4)	148 (83.6)	149 (84.2)	148 (83.1)	126 (70.8)	0.003
Diuretic	502 (70.7)	139 (78.5)	125 (70.6)	136 (76.4)	102 (57.3)	< 0.001
ACEI	19 (2.7)	3 (1.7)	3 (1.7)	7 (3.9)	6 (3.4)	0.43
Albumin infusion	60 (8.1)	12 (6.4)	15 (8.1)	16 (8.7)	17 (9.3)	0.76
ARB	87 (12.2)	14 (7.9)	15 (8.4)	28 (15.7)	30 (16.9)	0.011
Laboratory data						
Scr (**baseline)** μmol/L	88(71, 93.9)	88 (71, 88)	82.5 (71, 88)	86.9 (71, 88.9)	85.9 (71, 97)	0.14
Neut × 109/L	7.35(4.57, 11.79)	8.4 (4.7, 12.6)	9.0 (5.4, 13.3)	8.3 (4.7, 12.2)	5.8 (3.8, 9.7)	< 0.001
MCHC g/L	329 (321, 338)	327.0 (318.0, 337.0)	329.0 (321.5, 338.0)	326.0 (318.0, 336.5)	330.0 (322.0, 338.0)	0.009
WBC × 109/L	9.14 (6.20, 13.75)	9.57 (5.94, 14.32)	10.45 (6.41, 14.9)	9.82 (6.55, 14.54)	7.92 (5.61, 11.80)	0.004
RDW-SD fl	45.3 (42.0, 50.3)	46.5 (43.0, 51.0)	45.2 (42.1, 50.2)	45.5 (41.7, 50.75)	44.9 (42.3, 49.1)	0.062
BNP pg/ml	301.2 (115.3, 983.5)	308.8 (127.2, 994.4)	377.7 (144.7, 977.2)	402.0 (149.6, 1091.9)	176.6 (66.2, 613.1)	0.001
Glu mmol/L	6.63 (5.12, 9.41)	7.36 (5.28, 10.37)	7.04 (5.09, 10.29)	7.08 (5.21, 9.30)	6.18 (5.12, 8.38)	0.18
CRP mg/L	46.55 (11.32, 115.7)	97.6 (39.0, 221.6)	74.4 (18.9, 147.1)	45.6 (12.2, 97.1)	12.6 (3.7, 40.2)	< 0.001
Hb g/L	105 (86, 126)	92 (75.5, 108.5)	103.0 (85.0, 122.5)	110.0 (90.0, 129.0)	119.0 (96.0, 136.0)	< 0.001
Alb g/L	30.4 ± 6.39	22.3 ± 2.90	28.5 ± 1.27	32.6 ± 1.23	38.3 ± 3.37	< 0.001
TG	1.24 (0.88, 1.80)	1.22 (0.81, 1.68)	1.19 (0.86, 1.77)	1.16 (0.87, 1.70)	1.15 (0.86, 1.79)	0.73
TC	3.28 (2.42, 4.15)	2.23 (1.65, 3.22)	3.10 (2.27, 4.00)	3.42 (2.80, 4.03)	3.92 (3.31, 4.75)	< 0.001
ICU patients % (n)	431 (58.2)	109 (58.0)	122 (65.6)	114 (62.3)	86 (47)	0.002
CRRT %(n)	350 (47.3)	84 (44.7)	86 (42.6)	99 (54.1)	81 (44.3)	0.19
HA-AKI % (n)	211 (28.5)	39 (20.7)	53 (28.5)	55 (30.1)	64 (35)	0.023
90-day mortality %(n)	464 (62.7)	130 (69.1)	127 (68.3)	116 (63.4)	97 (49.7)	< 0.001
1- year mortality %(n)	488 (65.9)	136 (72.3)	133 (71.5)	124 (67.8)	95 (51.9)	< 0.001

*CI* Cerebral infarction, *PPI* Proton pump inhibitor (Omeprazole, Lansoprazole, Pantoprazole, Rabeprazole), *ACEI* ACEI Series Angiotensin Converting Enzyme Inhibitors, *ARB* Angiotensin II receptor antagonists, *Scr (baseline)* Normal creatinine values before the onset of AKI, *Neut* Neutrophils, *MCHC* Mean corpuscular hemoglobin concentration, *WBC* White blood cell count, *RDW-SD* Red blood cell volume distribution width, *BNP* Brain natriuretic peptide, *Glu* Blood glucose, *CRP* C-reactive protein, *ICU* Intensive Care Unit, *CRRT* Continuous renal replacement therapy

### ALB was associated with the 90-day and 1-year mortality of patients with AKI

Factors associated with mortality during follow-up were tested using Cox regression. In univariate analysis, Patients were older (*P* < 0.001), combined HF (*P* < 0.001), CHD (*P* = 0.003), cancer (*P* < 0.001), HA-AKI (*P* < 0.001), sepsis (*P* < 0.001), critical AKI (*P* < 0.001), lower baseline Scr (*P* = 0.002), higher Glu (*P* < 0.001), higher alkaline phosphatase (ALP) (*P* = 0.004), lower MCHC (*P* < 0.001), lower Hb (*P* = 0.005), higher CRP (*P* = 0.023), higher BNP (*P* < 0.001), higher RDW (*P* < 0.001) was independently associated with an increased risk of death. Moreover, Patients were treated in the ICU, and used proton pump inhibitors (PPI), angiotensin receptor blockers (ARB), diuretics (*P* < 0.001) was independently associated with an increased risk of death. When we used univariate Cox regression analysis with ALB as a continuous variable, we showed that for every 10 g/L increase in ALB, the 90-day and 1-year risk of death decreased by 29% (Table 2).

**Table 2:** Factors associated mortality in univariate analysis using Cox regression

Variable	90-day		1-year	
	HR (95% CI)	*P*-value	HR (95%CI)	*P*-value
Age (years)	1.02 (1.013, 1.024)	< 0.001	1.02 (1.015,1.026)	< 0.001
Sex, Male	1.07 (0.88, 1.30)	0.46	1.08 (0.89, 1.30)	0.41
DM	0.95 (0.76, 1.17)	0.63	0.95 (0.77, 1.17)	0.66
Hypertension	0.94 (0.78, 1.12)	0.50	0.96 (0.80, 1.15)	0.69
HF	1.80 (1.48, 2.19)	< 0.001	1.81 (1.49, 2.20)	< 0.001
CHD	1.59 (1.28, 1.97)	< 0.001	1.46 (1.13, 1.88)	0.003
Cancer	1.42 (1.14, 1.76)	0.001	1.49 (1.21, 1.83)	< 0.001
Sepsis	1.65 (1.37, 1.98)	< 0.001	1.63 (1.36,1.96)	< 0.001
CKD	0.79 (0.58, 1.07)	0.13	0.77 (0.57, 1.04)	0.099
Critical AKI	1.81 (1.40, 2.31)	< 0.001	1.86 (1.45, 2.40)	< 0.001
Scr(**baseline**)	0.995 (0.992, 0.998)	0.004	0.995 (0.991, 0.998)	0.002
Albumin /10	0.71 (0.61, 0.82)	< 0.001	0.71 (0.62, 0.81)	< 0.001
Glu	1.03 (1.01, 1.05)	< 0.001	1.03 (1.01, 1.05)	< 0.001
ALP	1.001(1.00, 1.002)	0.007	1.001(1.00, 1.002)	0.004
MCHC	0.99(0.98, 0.99)	0.001	0.99(0.98, 0.99)	< 0.001
WBC	1.01 (1.00, 1.02)	0.006	1.01 (1.00,1.02)	0.007
Neut	1.01 (0.99, 1.02)	0.079	1.01(0.99, 1.02)	0.079
Hb	0.996(0.993, 0.999)	0.024	0.995 (0.992, 0.999)	0.005
CRP	1.001(1.000, 1.002)	0.045	1.001(1.000, 1.002)	0.023
BNP	1.00 (1.00,1.00)	< 0.001	1.00 (1.00,1.00)	< 0.001
RDW	1.03(1.02, 1.04)	< 0.001	1.03 (1.02,1.04)	< 0.001
PPI	1.93 (1.46, 2.5)	< 0.001	1.87 (1.43, 2.44)	< 0.001
Diuretic	1.76(1.42, 2.26)	< 0.001	1.84 (1.47, 2.30)	< 0.001
ACEI	0.90(0.51, 1.61)	0.74	0.93 (0.53, 1.62)	0.81
ARB	0.56(0.40, 0.77)	0.001	0.58 (0.42, 0.79)	0.001
HA-AKI	1.67 (1.38, 2.02)	< 0.001	1.62 (1.34, 1.95)	< 0.001
ICU	2.96 (2.40, 3.65)	< 0.001	2.87 (2.35, 3.51)	< 0.001
Albumin infusion	1.27 (0.94, 1.71)	0.11	1.26 (0.94, 1.70)	0.12
CRRT	2.49 (2.06, 3.01)	< 0.001	2.38 (1.98, 2.96)	< 0.001

**Fig. 1:**
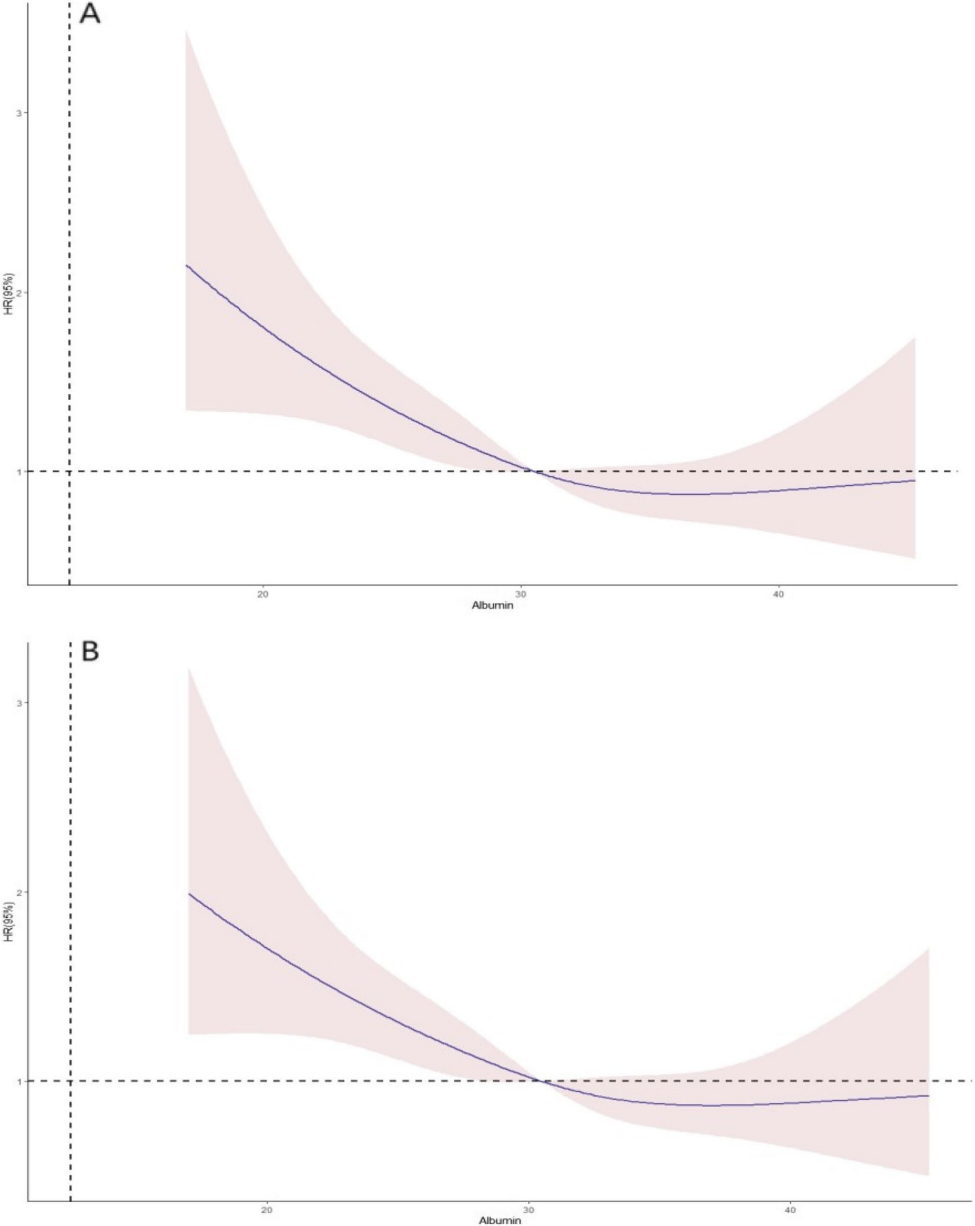
Association between Albumin and 90-day (**A**) and 1-year (**B**) all-cause mortality in AKI patients. Restricted cubic spline (RCS) regression models show a linear relationship between albumin and patient mortality, with solid purple lines representing a smooth curve fit between the variables. The pink areas represent 95% confidence intervals for the fit. All data were adjusted for age, sex, Critical AKI, CHD, Sepsis, cancer, HF, Scr (baseline), Glu, ALP, MCHC, Hb, CRP, RDW, WBC, BNP, HA-AKI, ICU, PPI, Diuretic and ARB, CRRT

After adjusting for confounders, we found a linear relationship between ALB and 90-day and 1-year mortality (*P* = 0.24), and that the risk of death in patients with AKI increased as ALB decreased (*P* = 0.001) (Fig. 1).

When ALB was used as a continuous variable, the higher the serum ALB level, the lower the mortality of patients with AKI. Compared with ALB Q4, the adjusted hazard ratio (HR) values for 90-day mortality was 1.76 (1.30, 2.38) in ALB Q1, 1.41 (1.05, 1.89) in Q2, and 1.12 (1.29, 0.83, 1.52) in Q3. The adjusted HR value for 1-year mortality was 1.79 (1.33, 2.41) in ALB Q1, 1.42 (1.06. 1.90) in Q2, and 1.20 (0.90, 1.61) in Q3. When ALB was used as a classification variable and ALB ≥ 34.8 g/L as a reference, it was found that when ALB ≤ 30.5 g/L, the 90-day and 1-year mortality of patients with AKI were substantially increased (as shown in Table 3).

**Table 3:** Multivariate logistic regression analysis for 90-day and 1-year mortality

ALP	Model 1	*P*	Model 2	*P*	Model 3	*P*	Model 4	*P*
90-day								
Quartile4	-							
Quartile1	1.82 (1.39, 2.38)	< 0.001	1.72 (1.31, 2.25)	< 0.001	1.49 (1.22, 1.97)	0.006	1.76(1.30, 2.38)	< 0.001
Quartile2	1.74 (1.32, 2.27)	< 0.001	1.61 (1.23, 2.11)	0.001	1.36 (1.03, 1.81)	0.029	1.41 (1.05, 1.89)	0.021
Quartile3	1.46 (1.11, 1.92)	0.006	1.33 (1.01, 1.75)	0.041	1.15 (0.86, 1.53)	0.33	1.12(1.29, 0.83, 1.52)	0.42
1-year								
Quartile4	-							
Quartile1	1.84 (1.42, 2.40)	< 0.001	1.74 (1.33, 2.26)	< 0.001	1.52 (1.15, 2.01)	0.003	1.79 (1.33, 2.41)	< 0.001
Quartile2	1.76 (1.35, 2.30)	< 0.001	1.62 (1.24, 2.11)	< 0.001	1.37 (1.04, 1.81)	0.023	1.42 (1.06. 1.90)	0.016
Quartile3	1.51 (1.16, 1.98)	0.002	1.37 (1.04, 1.79)	0.022	1.21 (0.91, 1.60)	0.18	1.20 (0.90, 1.61)	0.20

Model 1: Albumin < 34.5 g/L affected the mortality of patients within one year by univariate cox regression analysis

Model 2: Model 2 was adjusted for the gender and age

Model 3: Based on Model 2, Model 3 was adjusted for Critical AKI, CHD, Sepsis, cancer, HF, Scr (baseline), Glu, ALP, MCHC, Hb, CRP, RDW, WBC, BNP

Model 4: Based on Model 3, Model 4 was adjusted for HA-AKI, ICU, PPI, Diuretic and ARB, CRRT

**Fig. 2:**
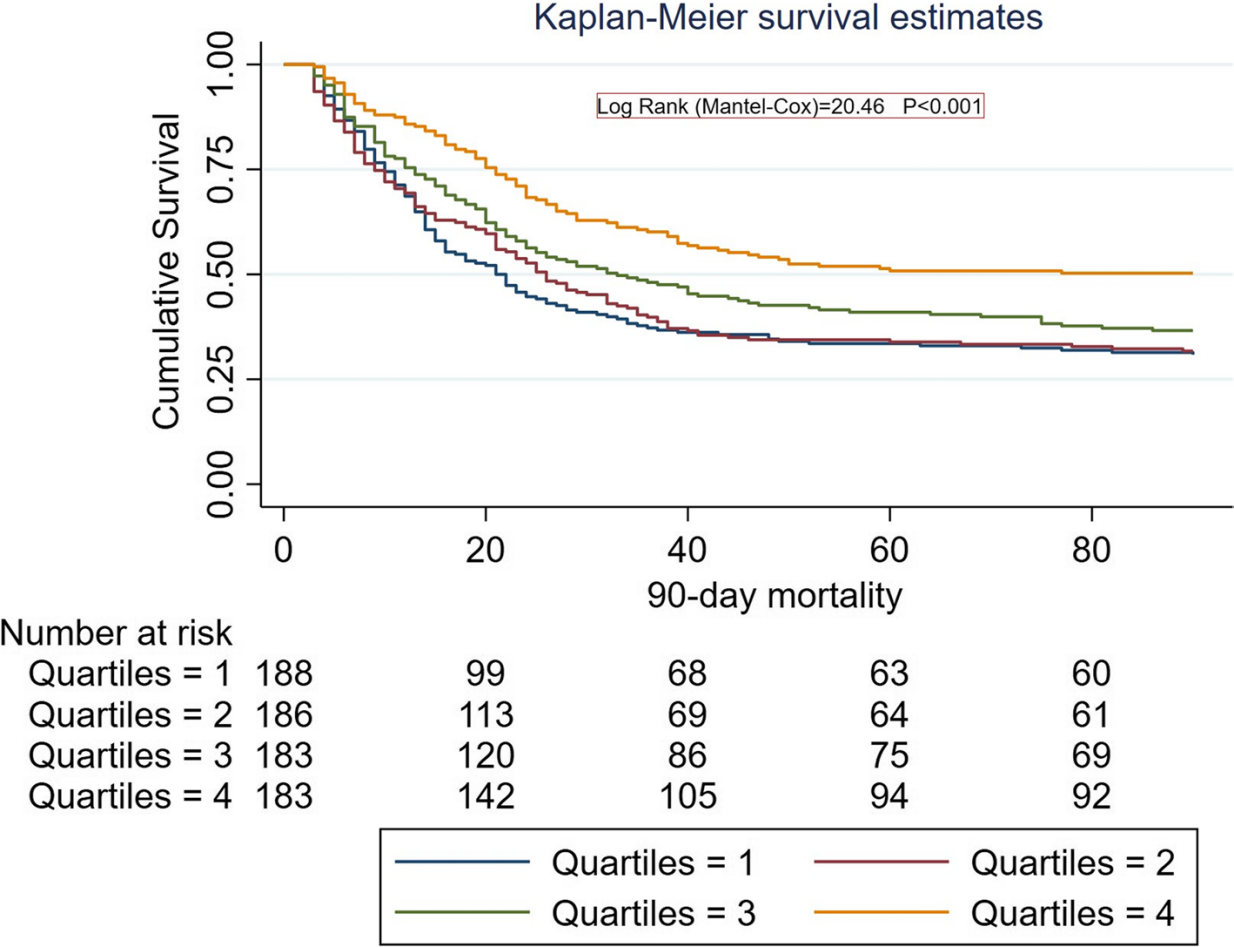
Survival curves for patients with different levels of serum ALB for 90-day

**Fig. 3:**
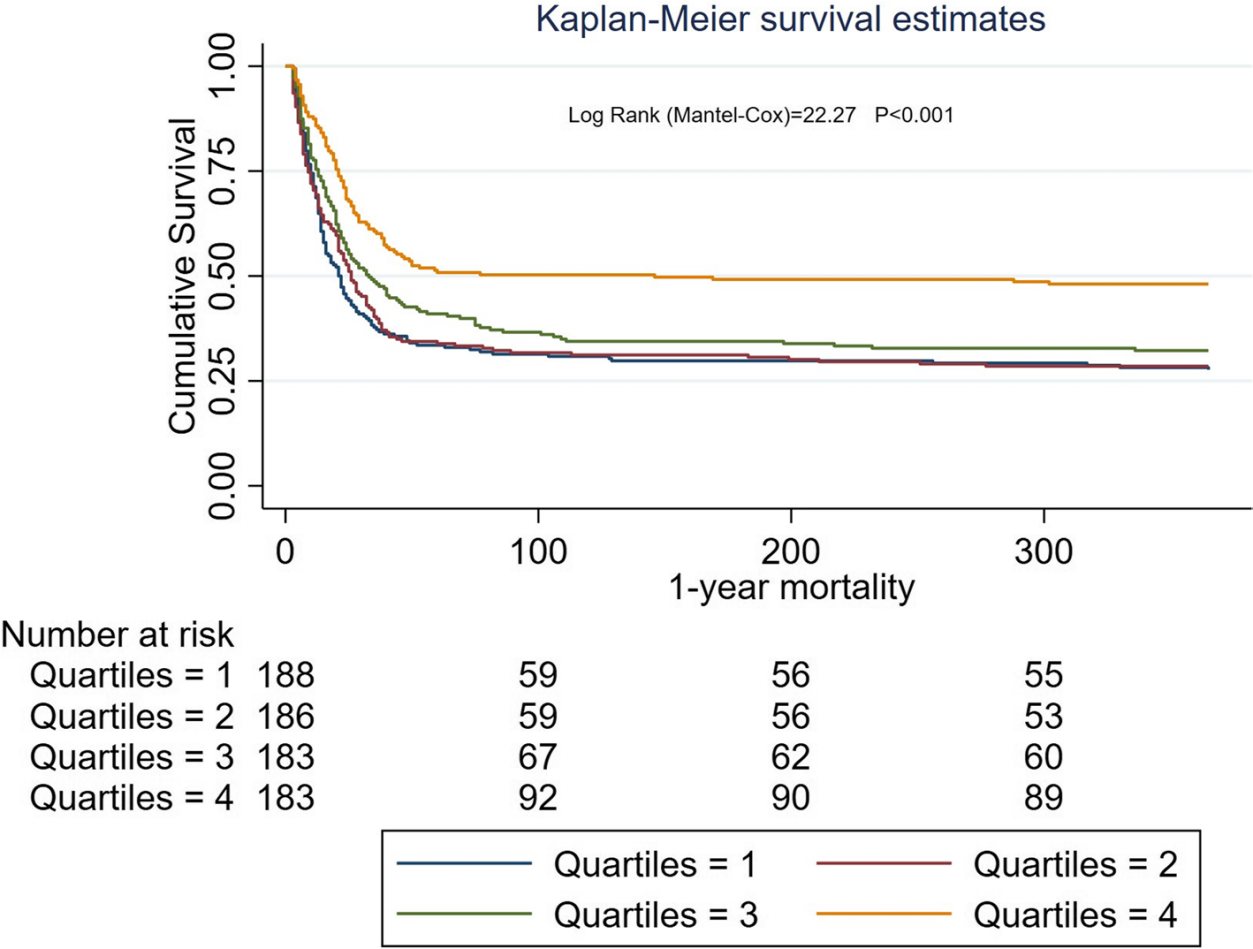
Survival curves for patients with different levels of serum ALB for 1-year

Kaplan–Meier curves show the association between ALB levels and mortality (90-day: log-rank = 20.46, *P* < 0.001; 1-year: log-rank = 22.27, *P* < 0.001) (Figs. 2 and 3).

**Fig. 4:**
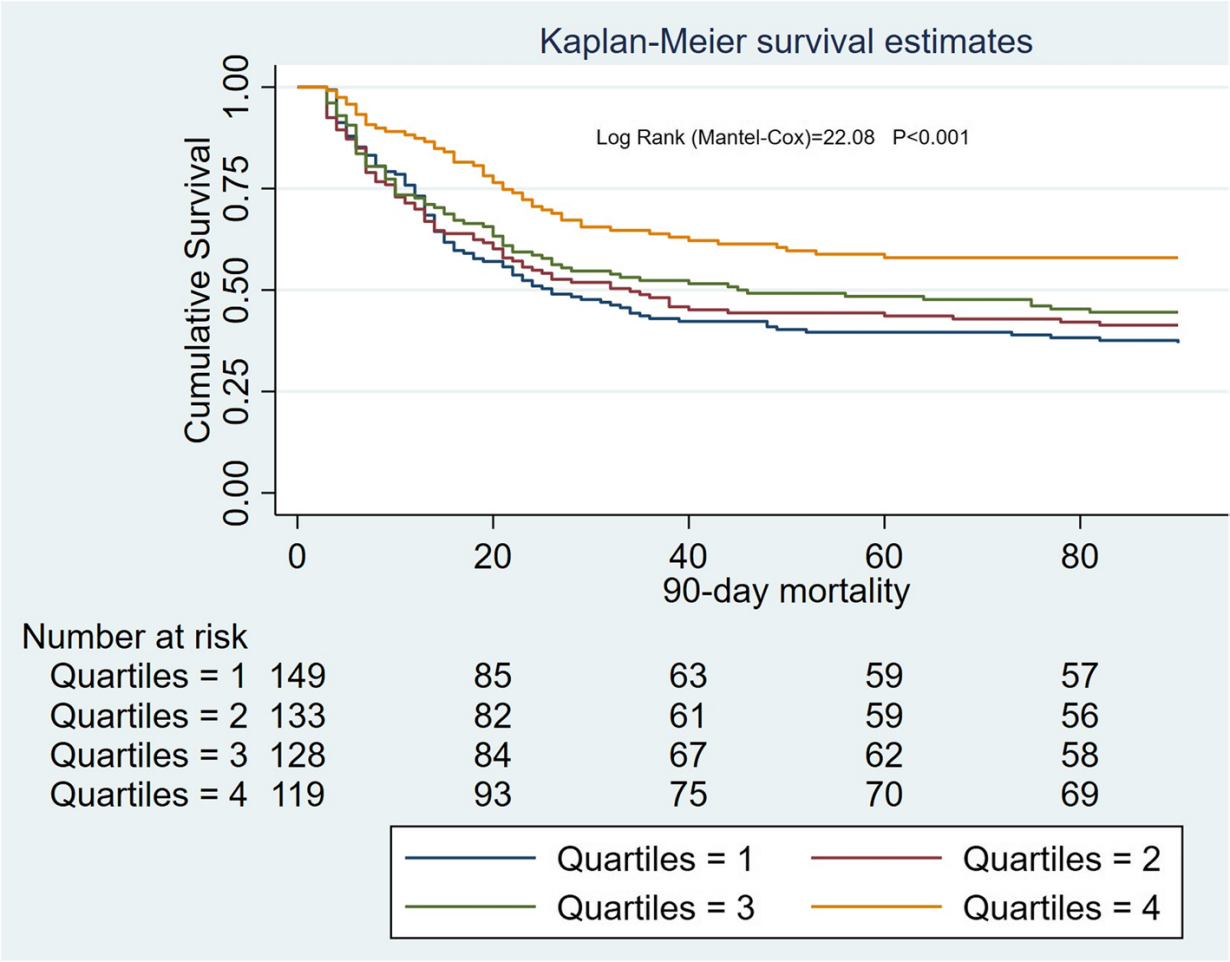
Survival curves for patients with different levels of serum ALB in community-acquired AKI

**Fig. 5:**
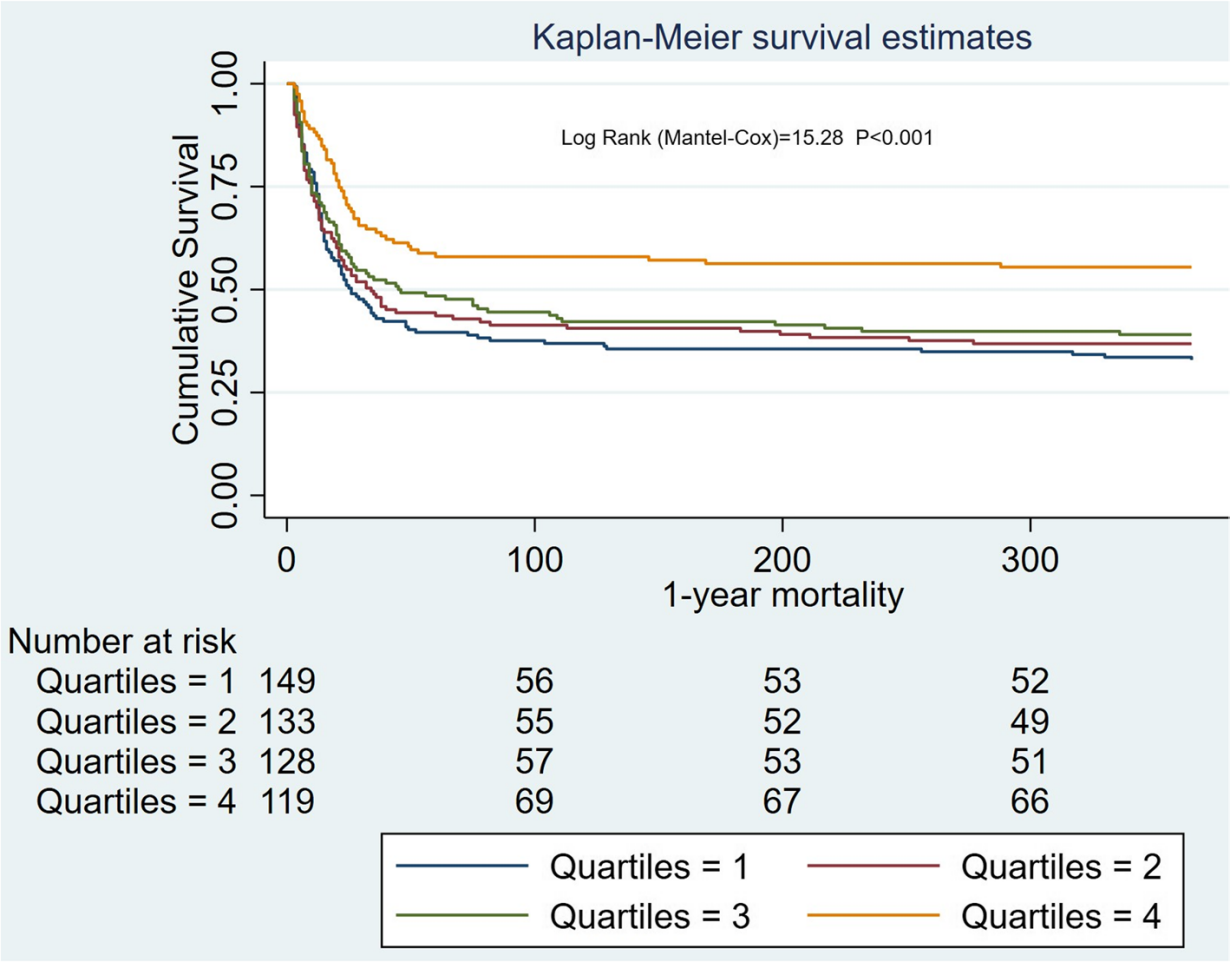
Survival curves for patients with different levels of serum ALB in community-acquired AKI

In patients with CA-AKI, Kaplan–Meier curves showed an association between ALB levels and mortality (90-day: log-rank = 22.08, *P* < 0.001; 1-year: log-rank = 15.28, *P* < 0.001 (Figs. 4 and 5).

**Fig. 6:**
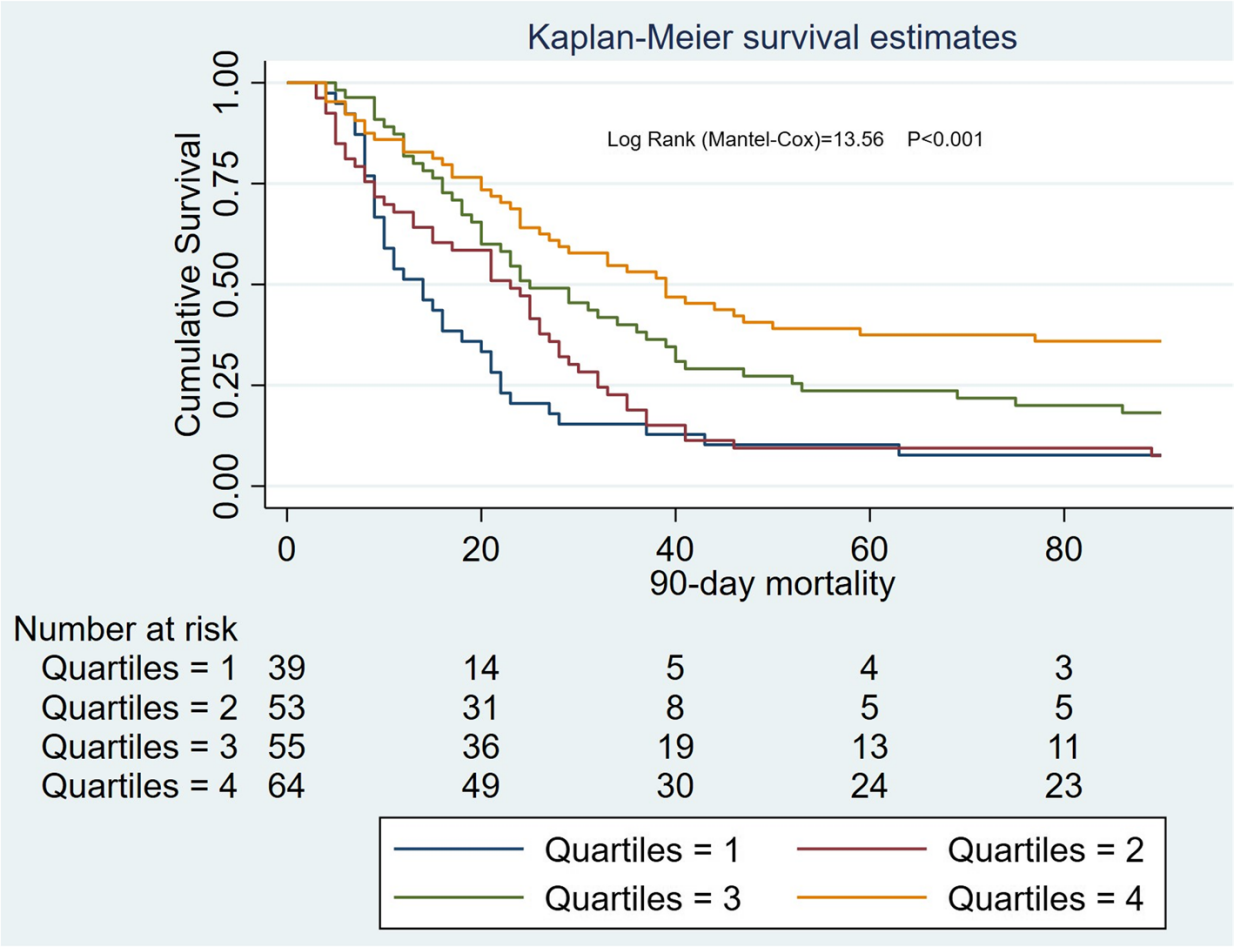
Survival curves for patients with different levels of serum ALB in hospital-acquired AKI

**Fig. 7:**
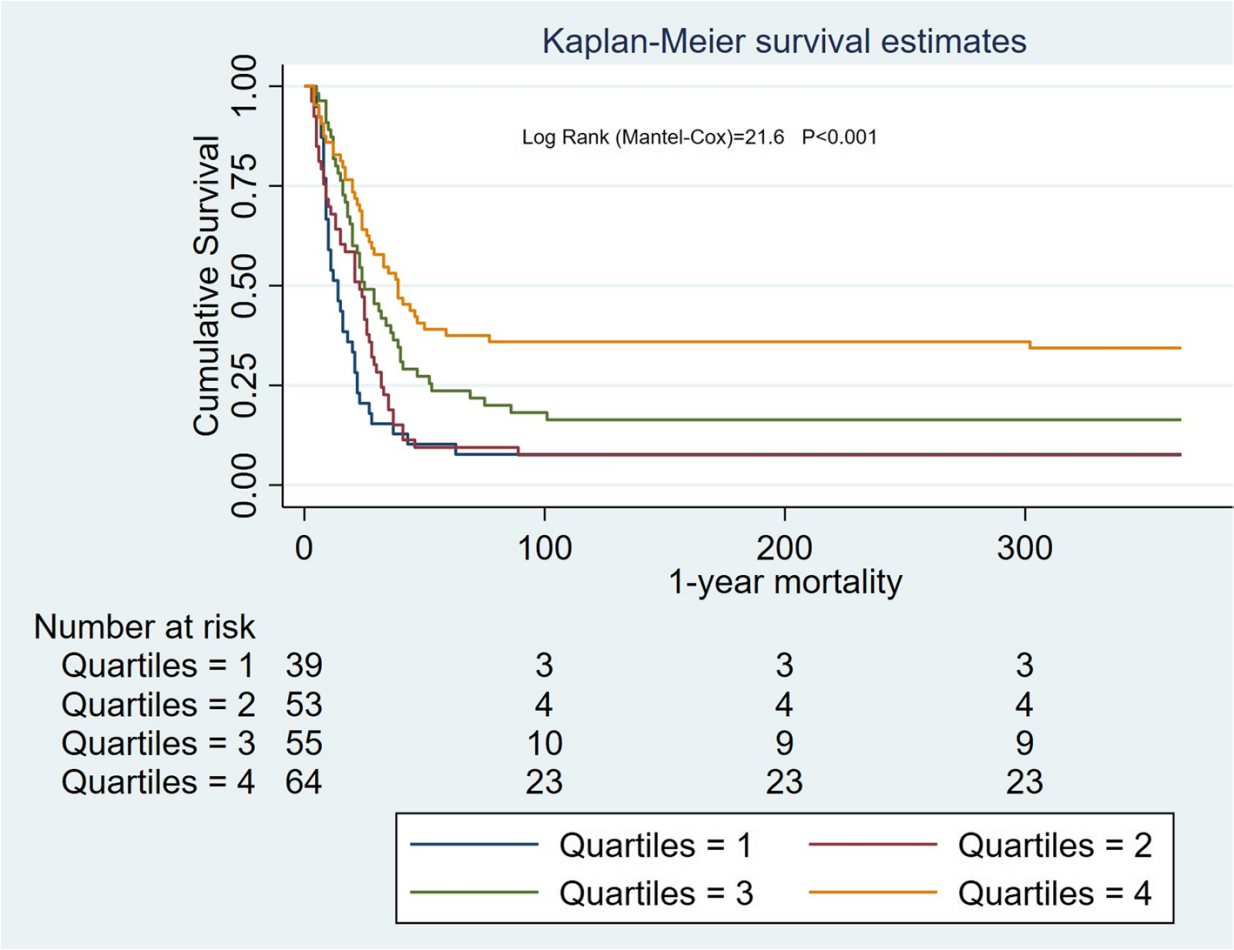
Survival curves for patients with different levels of serum ALB in hospital-acquired AKI

In patients with HA-AKI, Kaplan–Meier curves showed an association between ALB levels and mortality (90-day: log-rank = 13.56, *P* < 0.001; 1-year: log-rank = 21.6, *P* < 0.001 (Figs. 6 and 7).

## Discussion

The key finding of this analysis was that ALB levels are closely related to the prognosis of patients with AKI. The higher the ALB level, the lower the risk of all-cause mortality at 90-days and 1-year. Compared to patients with ALB ≥ 34.8 g/L, patients with 26.1 g/L < ALB ≤ 30.5 g/L have an increased risk of 90-day and 1-year all-cause mortality by approximately 40%, and patients with ALB ≤ 26.0 g/L have an increased risk of 90-day and 1-year all-cause mortality by approximately 76% and 79%, respectively.

ALB is an important protein in the human body that can maintain colloid osmotic pressure in blood vessels and maintain the water balance inside and outside the blood vessels. In addition, ALB has several vital functions, including carrying poorly water-soluble molecules, antioxidants, and anti-inflammatory effects, which have a protective effect on the kidneys [17–19]. Several factors can influence hypoalbuminemia, including inflammation, infection, malnutrition, protein-loss disorders, oxidative stress, cancer cachexia, and liver dysfunction [20–22]. Therefore, hypoalbuminemia may indicate the severity of the underlying disease and/or be a marker of malnutrition [23]. Our analysis also indicated that the lower the ALB level, the more patients with sepsis and the higher the inflammation indicators. Bovine serum albumin (BSA) reacts with nitrogen oxides to form S-nitroso-BSA, which can effectively dilate renal vessels for a long time and reduce renal ischemia and hypoxia [24]. In addition, hypoalbuminemia prevents the body from effectively removing toxic substances, resulting in reduced blood vessel volume and subsequent renal hypoperfusion, both of which can exacerbate kidney damage [25]. In recent years, a large number of studies have confirmed the correlation between the level of albumin and patient mortality [26]. Akirov et al. conducted a large retrospective study and found that significant hypoalbuminemia was associated with 34% mortality in hospitalized patients [27], and Thongprayoon et al. showed that even in the normal ALB range, the level of ALB was significantly correlated with the prognosis of patients [8]. A meta-analysis conducted by Wiedermann et al. [28] showed that a low ALB level is not only a risk factor for AKI but also a risk factor for death. Our study selected patients with AKI, and the results showed that the 1-year mortality rate decreased by 29% for each 10 g/L increase in ALB. After adjusting for multiple influencing factors, ALB levels remained independently correlated with patient prognosis.

Our study showed that ALB level had a significant impact on the prognosis of patients with CA-AKI and HA-AKI. In patients with CA-AKI, the ALB level at admission was the level at which AKI occurred. In patients with HA-AKI, the ALB level at admission was the same as that before AKI. Although our study suggests that ALB infusion before AKI does not have an impact on prognosis owing to the small number of patients receiving ALB infusion and the fact that the majority of patients refused ALB infusion due to economic constraints, the final analysis results are unreliable. Recent studies have shown that ALB infusion can improve the discharge rate of patients with AKI with sepsis and is associated with a decrease in the 28-day mortality [29, 30]. Our research shows that patients with high ALB levels appear to have a better prognosis; however, further research is needed to confirm whether ALB infusion can improve patient prognosis in clinical practice.

Elevated creatinine levels are associated with poor prognosis in patients [31], but our univariate Cox regression analysis indicates that a high baseline level of creatinine is associated with a good prognosis. However, except for a few patients with CKD, the baseline values of most patients were normal. Creatinine is an index reflecting the content of human muscle tissue [32]; most patients with high creatinine levels in the normal range are fitness workers or male. Therefore, it may be beneficial for healthy individuals to have a high level of creatinine within the normal range. However, after adjusting for various confounding factors, we found that the baseline value of creatinine had no statistically significant impact on prognosis.

This study had some limitations. First, this was an observational, retrospective, single-center study, which may have introduced a selection bias. This leads to a possible risk of bias in this research. In addition, we do not have a large sample study on whether infusion of albumin improves the prognosis of patients with AKI. Therefore, the conclusion needs to be verified through a prospective study.

## Conclusion

We analyzed the clinical data of 740 patients in the electronic medical record system of Zhejiang Provincial People's Hospital. Our results showed that a serum albumin < 30.5 g/L was associated with an increased risk of 90-day and 1-year all-cause mortality. However, whether can improve outcomes by increasing the ALB level in hospitalized patients requires further investigation.

## Data Availability

The datasets used and/or analyzed in the current study are available from the corresponding author upon reasonable request.

## Abbreviations

ALB: Albumin
AKI: Acute kidney injury
CI: Cerebral infarction
PPI: Proton pump inhibitor
ACEI: Angiotensin converting enzyme inhibitors
ARB: Angiotensin receptor blockers
MCHC: Mean corpuscular hemoglobin concentration
WBC: White blood cell count
RDW-SD: Red cell distribution width-standard deviation
BNP: Brain natriuretic peptide
CRP: C-reactive protein
ICU: Intensive care unit
CRRT: Continuous renal replacement therapy
CKD: Chronic kidney disease
KDIGO: Kidney Disease: Improving Global Outcomes
CHD: Coronary atherosclerotic heart disease
HF: Heart failure
COPD: Chronic obstructive pulmonary disease
HA-AKI: Hospital-acquired-AKI
CA-AKI: Community-acquired-AKI
Scr: Serum creatinine
MDRD: Modification of diet in renal disease
SD: Standard deviation
IQR: Interquartile distance
ANOVA: Analysis of variance
HR: Hazard ratios
CI: Confidence intervals
RCS: Restricted cubic spline
BSA: Bovine serum albumin
